# The Role of PET-Based Radiomic Features in Predicting Local Control of Esophageal Cancer Treated with Concurrent Chemoradiotherapy

**DOI:** 10.1038/s41598-018-28243-x

**Published:** 2018-07-02

**Authors:** Junfeng Xiong, Wen Yu, Jingchen Ma, Yacheng Ren, Xiaolong Fu, Jun Zhao

**Affiliations:** 1 School of Biomedical Engineering, Shanghai Jiao Tong University, Shanghai, China; 2 Department of Radiation Oncology, Shanghai Chest Hospital, Shanghai Jiao Tong University, Shanghai, China

## Abstract

This study was designed to evaluate the predictive performance of ^18^F-fluorodeoxyglucose positron emission tomography (PET)-based radiomic features for local control of esophageal cancer treated with concurrent chemoradiotherapy (CRT). For each of the 30 patients enrolled, 440 radiomic features were extracted from both pre-CRT and mid-CRT PET images. The top 25 features with the highest areas under the receiver operating characteristic curve for identifying local control status were selected as discriminative features. Four machine-learning methods, random forest (RF), support vector machine, logistic regression, and extreme learning machine, were used to build predictive models with clinical features, radiomic features or a combination of both. An RF model incorporating both clinical and radiomic features achieved the best predictive performance, with an accuracy of 93.3%, a specificity of 95.7%, and a sensitivity of 85.7%. Based on risk scores of local failure predicted by this model, the 2-year local control rate and PFS rate were 100.0% (95% CI 100.0–100.0%) and 52.2% (31.8–72.6%) in the low-risk group and 14.3% (0.0–40.2%) and 0.0% (0.0–40.2%) in the high-risk group, respectively. This model may have the potential to stratify patients with different risks of local failure after CRT for esophageal cancer, which may facilitate the delivery of personalized treatment.

## Introduction

Esophageal cancer is the sixth most common cause of cancer-related death worldwide^1^. China has a particularly high rate of esophageal cancer, with the highest prevalence and mortality rates in the world, especially for esophageal squamous cell carcinoma (ESCC). Concurrent chemoradiotherapy (CRT) has become the current standard treatment for locally advanced esophageal cancer since RTOG 85-01. However, even with the current treatment, the prognosis for patients with unresectable esophageal cancer is poor, and local failure after definitive CRT continues to follow the primary failure pattern^2^. Future therapeutic strategies should focus on enhancing local control, and potential factors that could predict the risk of local failure may help physicians deliver personalized treatment to patients with different risks. For example, radiation dose escalation could be delivered to those with a higher risk of local relapse.

In addition to the frequently used patient-based clinical factors, including TNM stage and tumor size, ^18^F-fluorodeoxyglucose positron emission tomography (^18^F-FDG PET) image-derived parameters, such as the Standardized Uptake Value (SUV), metabolic tumor volume (MTV), and total lesion glycolysis (TLG), have been reported to be useful in predicting the pathological response to neoadjuvant CRT or determining the prognosis of patients with esophageal cancer^3–15^. However, predicting treatment outcomes with a single traditional feature, usually correlated with SUV_max_ or SUV_mean_, has two considerable limitations: 1) Tumors show significant heterogeneity in both the range and spatial distribution of 18F-FDG uptake^5,6,8^, which cannot be represented by a single point (SUV_max_) or a mean value (SUV_mean_); and 2) the noise of the PET image may result in inaccurate estimation of a single point^7^. Therefore, to improve the prediction accuracy, it is necessary to explore additional features to describe the heterogeneity within a tumor and determine the underlying correlation between these features and treatment outcomes.

In recent years, radiomics analysis has been widely studied in lung, breast, prostate, and head-and-neck cancer^16–22^ as a tool that converts medical images into mineable data by the high-throughput extraction of quantitative features, which may potentially improve diagnostic, prognostic, and predictive accuracy. Therefore, we hypothesized that radiomic features could help in predicting treatment outcomes in patients with esophageal cancer.

In the present study, we proposed and compared models of ^18^F-FDG PET-based radiomic features with or without clinical features using four machine-learning methods [random forest (RF)^23,24^, support vector machine (SVM)^25^, logical regression (LR), and extreme learning machine (ELM)^26^] to predict local control in patients with esophageal cancer treated with CRT.

## Related Works

Recently, radiomics analysis has been widely used in diagnosis and treatment response prediction. Lambin *et al*.^11^ described radiomics as a bridge between medical imaging and personalized medicine. Fehr *et al*.^12^ proposed using MRI-based texture features to automatically classify prostate cancer using Gleason scores. Coroller *et al*.^13^ found radiomic phenotype features were predictive for pathological response in non-small cell lung cancer. Maforo *et al*.^16^ investigated computer-extracted tumor phenotypes by using radiomic features extracted from diffusion-weighted imaging. Aerts *et al*.^19^ used the radiomics approach to decode tumor phenotypes by noninvasive imaging. Zhao *et al*.^5^ reported that intratumoral ^18^F-FDG distribution corresponds well to the expression levels of Glut-1, Glut-3, and HK-II. Tixier F. *et al*.^6^ proposed several textural features to predict the therapy response in esophageal cancer and demonstrated that these features of tumor metabolic distribution allowed the best stratification of esophageal carcinoma patients in the context of therapy response prediction. Tan *et al*.^8^ tried to use spatial-temporal ^18^F-FDG-PET features to predict the pathologic response of esophageal cancer to neoadjuvant chemoradiation therapy. Moreover, many studies have focused on the changes in radiomics features (delta-radiomics features), and found their potential prognostic value in cancer. Fave *et al*.^14^ reported that the delta-radiomics features calculated from CT images can be used to predict the patient outcomes in non-small cell lung cancer. Cunliffe *et al*.^15^ utilized delta-radiomics features to identify patients who would develop radiation pneumonitis during treatment in esophageal cancer.

Many machine-learning methods can be used to identify the radiomic features predicting the local control status, such as RF, SVM, LR, and ELM^23,25,26^. The RF classification method is robust against overfitting and contains several decision trees. Each decision tree generates a prediction and the final result is determined by accumulating the votes of all decision trees. The SVM model uses an RBF kernel to map training samples into a high-dimensional space, and aims to find a hyper-plane that can linearly separate the two classes (local control and local failure) by the widest margin. The LR model first uses a logit function to transform training samples to make the corresponding output values fall within a range (usually [0–1]). Then, a linear function is used to approximate the transformed samples. ELMs are feedforward neural networks for classification and regression with a single layer or multiple layers of hidden nodes, and the parameters of the hidden nodes does not need to be tuned.

## Materials and Methods

### Patient Population

This study comprised 30 ESCC patients who were prospectively included in a clinical trial (NCT01843049) and were treated with definitive concurrent CRT between April 2012 and June 2015^27^. The study was approved by the local institutional review board at Shanghai Chest Hospital, Shanghai, China. All patients were required to provide informed consent at the time of enrollment. We confirmed that all methods were performed in accordance with the relevant guidelines and regulations.

### PET Imaging

Patients underwent PET/CT scans both before CRT (pre-CRT) and after receiving 20 fractions of radiation doses (mid-CRT). PET/CT scans were obtained with a BIOGRAPH 16HR (Siemens Molecular Imaging, Knoxville, TN) with an axial field of view of 16.2 cm. All images were composed of 128 × 128 pixels with voxel dimensions of 4 × 4 × 4 mm. Patients fasted for at least 6 h before the administration of 0.2 mCi/kg or 7.4 MBq/kg body weight of 18F-FDG, which was produced onsite using Siemens Cyclotron (Eclipse ST 111, Siemens/CTI, Knoxville, TN), and they then rested for approximately 60 (range, 55–75) minutes before the PET scan. The 16-slice CT process was performed for attenuation correction with an X-ray tube voltage and current peak of 120 kV and 120 mA, respectively, a slice thickness of 5 mm, and a spacing of 4 mm. The images were reconstructed using a three-dimensional ordered subset expectation maximization algorithm and attenuation correction derived from the CT data.

### Treatment and Follow-Up

All patients were treated with definitive concurrent CRT. The definition and dose prescription of radiotherapy target volumes and dose constraints to the organs at risk followed the protocol of our previous study on dose escalation^27^.

Patients received 2 cycles of concurrent chemotherapy (cisplatin 25 mg/m^2^ IV daily on days 1–3 and 29–31 plus 5-Fluorouracil (5-FU) 500 mg/m^2^ IV daily via continuous infusion over 24 h on days 1–4 and 29–32) during the radiotherapy period. Consolidation chemotherapy (cisplatin 25 mg/m^2^ IV daily on days 1–3 plus 5-FU 600 mg/m^2^ IV daily on days 1–5, cycled every 4 weeks) was given for 2 cycles 1 month after the end of concurrent CRT.

Follow-up evaluations were performed 1 month after the completion of all treatments, then every 3 months for 2 years and then every 6 months thereafter. Each evaluation included a physical examination, a blood test, barium esophagram, CT scan of the neck and chest, and abdominal ultrasound; endoscopy and biopsy were performed once local recurrence was suspected.

### Delineation of Volumes of Interest

For each patient, a tumor volume of interest (VOI) was defined on pre-CRT PET images by two experienced radiation oncologists who reviewed all available resources, including diagnostic CT, 18F-FDG PET/CT, barium esophagography, and endoscopic reports, and then reached a consensus on the contouring. VOIs contained the primary tumors only and excluded the involved lymph nodes because it has been found that quantitative features extracted from small lesions (particularly those less than 5 mL) yield less reproducible and consistent results compared with those from larger tumors^25,28^. The mid-CRT PET images of each patient were then registered with the corresponding pre-CRT images, and the contour of the VOI was projected onto the mid-CRT images. VOIs and their corresponding images were imported into MATLAB for further analysis.

### Feature Extraction and Selection

#### Radiomic Features

Quantitative radiomic features of four categories were extracted from VOIs, with SUV computed based on body weight: 14 first-order statistics, 8 shape- and size-based features, 34 textural features, and 384 wavelet features^19^. Table 1 shows the details of these features. A total of 440 features were obtained from one set of PET images for each VOI.

1. *14 first-order statistics:* these features describe the distribution of ^18^F-FDG uptake. They are calculated based on the three-dimensional PET image matrix *X* with *N* voxels and the first-order histogram *P* with ${N}_{g}$ discrete intensity levels.
2. *8 shape- and size-based features:* these features describe the geometric characteristics of a tumor. They are computed based on the volume *V* and the surface area *A* of the VOI.
3. *34 textural features:* these features describe the patterns or the spatial distribution of voxel intensities. Different from the features mentioned above, which are related to the gray-level distribution of a PET image, the textural features are calculated from a gray-level co-occurrence matrix (GLCM) with a bin size of 0.1 and gray-level run-length matrix (GLRLM) texture matrices with a bin size of 0.1.
4. *384 wavelet features:* wavelet transform uses “coiflet 1” filters to decompose the original image in low frequencies and high frequencies in a similar manner as Fourier analysis, which can decouple textural information. Specifically, the original three dimensional PET image (*X*) is decomposed into eight decompositions, i.e., ${X}_{{HHH}}$, ${X}_{{HHL}}$, ${X}_{{HLH}}$, ${X}_{{HLL}}$, ${X}_{{LHH}}$, ${X}_{{LHL}}$, ${X}_{{LLH}}$, and ${X}_{{LLL}}$, where *H* and *L* indicate high-pass and low-pass functions, respectively. Mathematically, ${X}_{{HLH}}$ is calculated by a formula and results from directional filtering with a high-pass filter along the *x* direction, a low-pass filter along the *y* direction and a high-pass filter along the *z* direction.

1 $${X}_{HLH}(i,j,k)={\sum }_{n=1}^{{R}_{H}}\,{\sum }_{m=1}^{{R}_{L}}\,{\sum }_{r=1}^{{R}_{H}}\,H(n)L(m)H(r)X(i+n,j+m,k+r)$$

where *X* is the original image. ${R}_{H}$ and ${R}_{L}$ are the lengths of filters *H* and *L*, respectively. The other decompositions are formulated in the same way. After eight decompositions are obtained, each decomposition is used as an input image to calculate the *first-order statistics* and the *textural features* as mentioned above.

**Table 1:** The detailed radiomic features.

***Radiomic Features***
***14 first order statistics:*** Energy, entropy, kurtosis, maximum, mean, mean absolute deviation, median, minimum, range, root mean square, skewness, standard deviation (Std), uniformity, variance.
***8 shape- and size-based features:*** Compactness 1, compactness 2, maximum 3D diameter, spherical disproportion, sphericity, surface area, surface to volume ratio, volume.
***34 textural features:*** Autocorrelation, cluster prominence, cluster shade, cluster tendency, contrast, correlation, difference entropy, dissimilarity, difference variance, energy_c, entropy_c, homogeneity 1, homogeneity 2, informational measure of correlation 1 (IMC1), informational measure of correlation 2 (IMC2), inverse difference moment normalized (IDMN), inverse difference normalized (IDN), inverse variance, maximum probability, sum average, sum entropy, sum variance, variance, short run emphasis (SRE), long run emphasis (LRE), gray-level non-uniformity (GLN), run length non-uniformity (RLN), run percentage (RP), low gray-level run emphasis (LGLRE), high gray-level run emphasis (HGLRE), short run low gray-level emphasis (SRLGLE), short run high gray-level emphasis (SRHGLE), long run low gray-level emphasis (LRLGLE), long run high gray-level emphasis (LRHGLE).
***384 wavelet features:*** Wavelet features consist of the first order statistics and textural features extracted from eight wavelet decompositions (${X}_{{HHH}}$, ${X}_{{HHL}}$, ${X}_{{HLH}}$, ${X}_{{HLL}}$, ${X}_{{LHH}}$, ${X}_{{LHL}}$, ${X}_{{LLH}}$, and ${X}_{{LLL}}$). For example, Energy_HHL represents the energy feature calculated from decomposition ${X}_{{HHL}}$.

#### Clinical and demographic features

Eight clinical and demographic features were incorporated into the analysis, including tumor location, TNM stages, radiation therapy doses, cycles of chemotherapy, age, and gender.

#### Feature Selection

Receiver operating characteristic (ROC) analysis was performed to determine the performance of the radiomic features in predicting the local control status, and the area under curve (AUC) was calculated. Features with higher AUC values were selected as discriminative radiomic features. The number of selected features was tuned form 10 to 400 with step size of 5.

### Predictive Model Construction

Four machine-learning methods were used, including random forest (RF), support vector machine (SVM), logistic regression (LR), and extreme learning machine (ELM), to construct predictive models. According to the last follow-up status, patients who experienced local progression were defined as positive samples (label is 1) and were regarded as a high-risk group. The others were defined as negative samples (label is 0) and assigned into a low-risk group. Five groups of features were respectively fitted in different models, including clinical features, pre-CRT radiomic features, mid-CRT radiomic features, all radiomic features, and all features.

#### Random Forest

The RF classification method, which is robust against overfitting, was adopted to combine the merits and ignore the weaknesses of selected features. In each fold of cross validation, 100 decision trees were grown. For each node of the tree, *p* samples were randomly drawn with replacement from the original data and *q* candidate features were randomly selected from input features. We used the grid searching method to tune the two parameters of RF, the samples (*p*) and candidate features (*q*) of each tree. The value of *p* was varied from 5 to 20 with a step size of 5 and the *q* value was similarly tuned from 5 to 50 with a step size of 5. In the testing step, each decision tree generated a prediction for the local control status of a patient, and the risk score for this patient was determined by the percentage of the number of trees voting for local failure among all decision trees. Patients with risk score greater than 0.5, who were more likely to experience local progression, were classified into high-risk group, and those with risk score less than 0.5 were classified into low-risk group.

#### Support Vector Machine, Logistic Regression, and Extreme Learning Machine

For comparison with RF, we also implemented the widely used SVM, LR, and ELM models.

SVM models map training samples into a high-dimensional space and find hyper-planes that can linearly separate the samples by the widest margin. We used the grid searching method to tune the two parameters of SVM with a radial basis function (RBF) kernel, the penalty factor *C* and the ${\rm{\sigma }}$ of the RBF kernel. The value of *C* was varied around ${10}^{t}$, where *t* ranged from −5 to 5 with a step size of 1. The ${\rm{\sigma }}$ was tuned from 0.1 to 1 with a step size of 0.1. Given a test patient, the output of the model was the “distance” of the specific patient to the optimized hyper-plane. Patients with the distance >0 would be regarded as local control and classified into low-risk group, and those with the distance ≤0 would be regarded as local progression and classified into high-risk group.

The LR model first used a logit function to transform the training samples to make the corresponding output values fall within a range (usually [0–1]). Then, a linear function was used to approximate the transformed samples. In the testing step, a linear predictor would be generated by the LR model for each patient, which was the risk score. Patients with risk score greater than 0.5, who were more likely to experience local progression, were classified into high-risk group, and those with risk score less than 0.5 were classified into low-risk group.

ELM is defined as a single-hidden layer feedforward neural network, and it randomly chooses hidden nodes and analytically determines the output weights of the feedforward neural networks. We used the grid searching method to tune the number of hidden nodes of the ELM with sigmoidal or sine functions, ranging from 5 to 30 with a step size of 5. Our ELM had two output nodes, and a test patient would be regarded as local control and classified into the low-risk group if the value of the first node was bigger than the second one, otherwise the patient would be regarded as local progression and classified into the high-risk group.

### Statistical analysis

**Figure 1:**
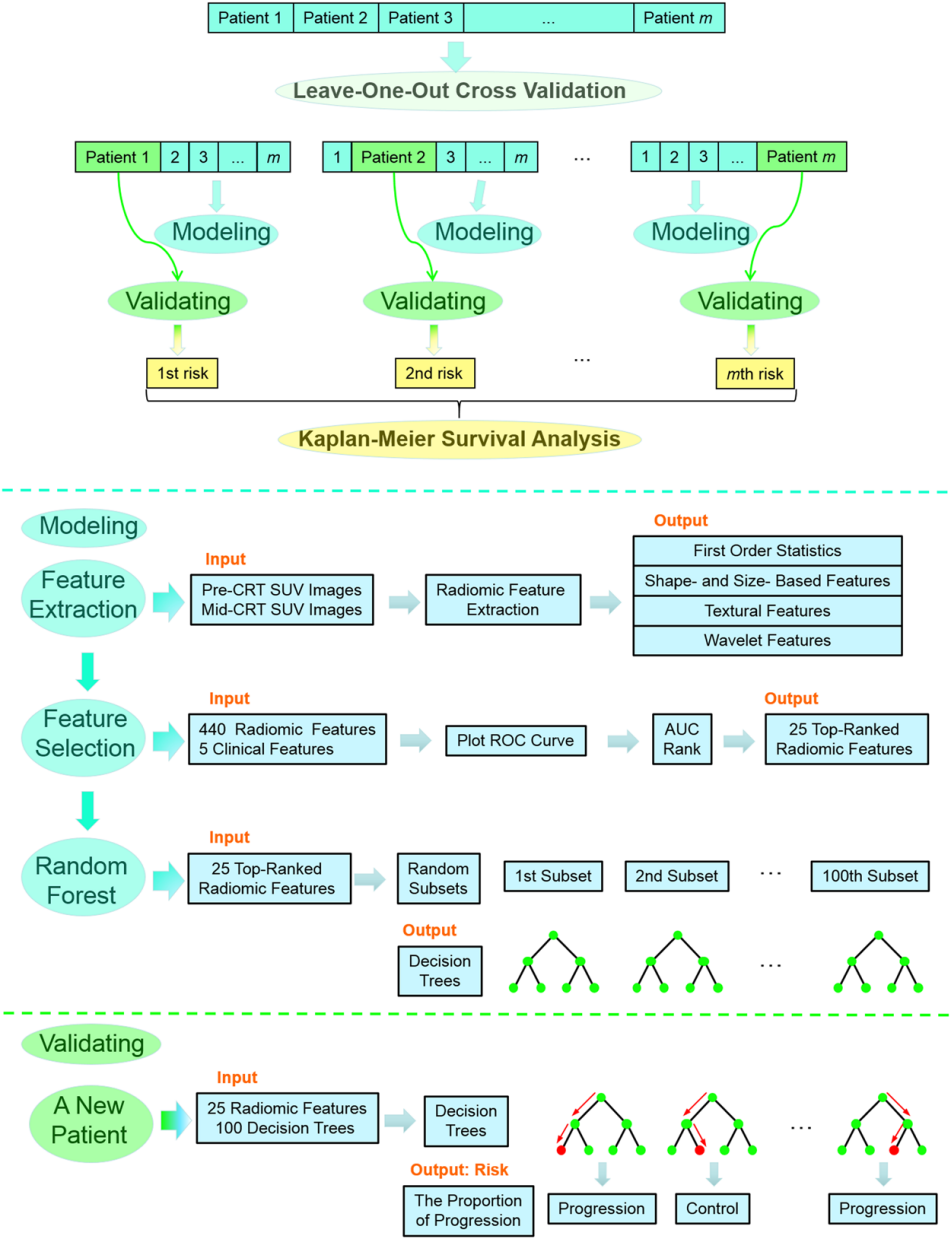
The workflow of the development and cross validation of the RF model developed with radiomic features.

The leave-one-out cross validation was adopted, which tests patient *i* in the model developed from the remaining cohort when *i* is left out. Specifically, for each cross validation fold, the *i*-th patient is used for testing and the remaining patients (except for *i*-th patient) are used to perform feature selection and the classifier construction. Figure 1 shows the workflow of the RF model development and validation with clinical features and radiomic features extracted from both pre- and mid-CRT images.

As mentioned in the *Predicting Models Construction* section, we constructed 20 models and evaluated their performances for the prediction of local control status. Each model was built by a classifier (RF, SVM, LR, or ELM) involving a specific group of features (clinical features, pre-CRT radiomic features, mid-CRT radiomic features, all radiomic features, or all features). Then, the patients were classified into low- and high-risk groups according to the model with the best predictive performance. The separation of Kaplan-Meier curves in terms of the local control rate and PFS was evaluated between the two groups using the log-rank test. The significance level was set at p < 0.05.

## Results

The characteristics of the 30 enrolled patients are shown in Table 2. The median follow-up time was 18.5 (range, 2.5~46.5) months.

**Table 2:** The characteristics of the patients.

Characteristics	Number of patients (n = 30)
Gender	
Male	26
Female	4
Age, years	
Median	63
Range	44–75
Performance status (ECOG)	
0	16
1	14
Tumor location	
Cervical	1
Upper thoracic	10
Middle thoracic	12
Lower thoracic	7
T stage (UICC 2002)	
T1/T2	4
T3	20
T4	6
N stage (UICC 2002)	
N0	11
N1	19
M stage (UICC 2002)	
M0	23
M1	7
Cycles of chemotherapy	
1 cycle	2
2 cycles	3
3 cycles	8
4 cycles	17
Biological equivalent dose (Gy)	
67.200	4
71.175	12
73.925	14
Status of local control	
Local progression	7
Local control	23

**Figure 2:**
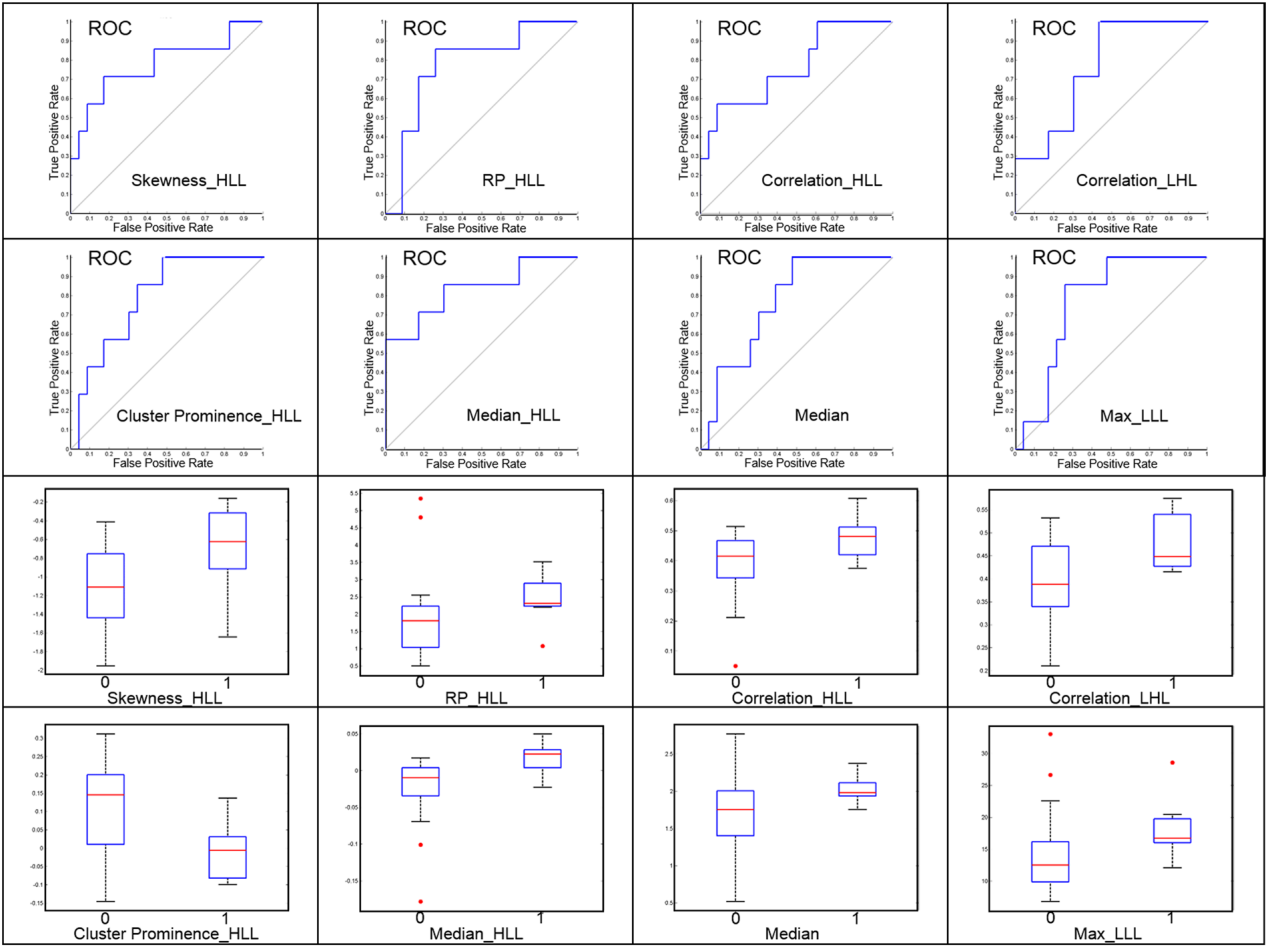
Performance of typical discriminative radiomic features for determining the local control of esophageal cancer after CRT. The upper two rows show ROC curves based on features extracted from pre- and mid-CRT SUV images, respectively. The lower two rows show the values of the features extracted from pre- and mid-CRT SUV images plotted against local control status, with 1 on the horizontal axis representing local progression and 0 representing local control, respectively.

**Figure 3:**
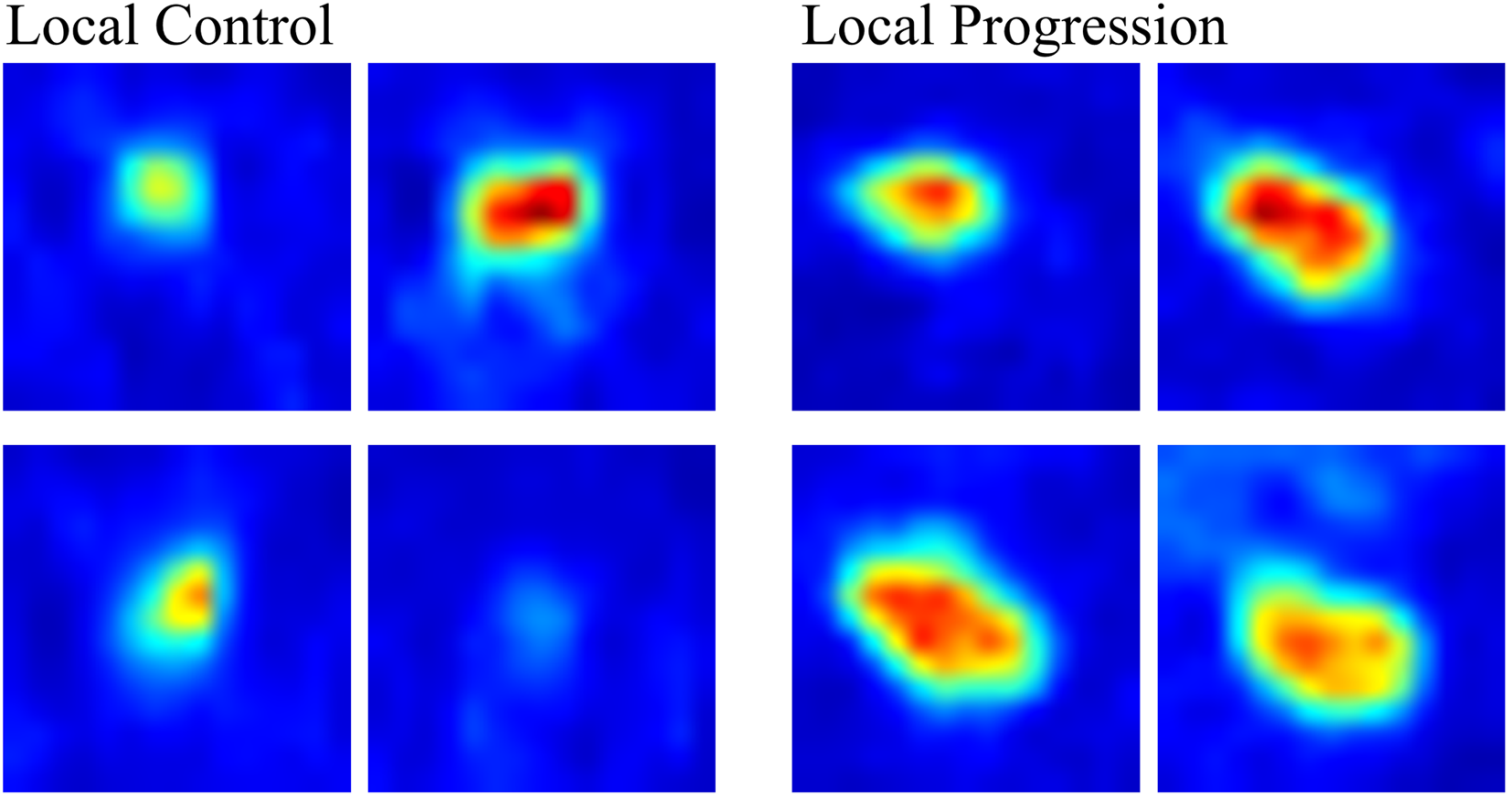
PET images of two typical patients with local control (left) and local progression (right), respectively. The skewness_HLL, RP_HLL, cluster promience_HLL, and median_HLL values are 0.164, −1.744, 65280, and −0.038 for the patient with local control and 0.743, −0.278, 24240, and 0.003 for the patient with local progression.

Among the 440 radiomic features derived from both pre- and mid- CRT PET images, 25 features with the highest AUCs for discriminating the local control status were selected from each fold of the cross validation. Then, the 25 features that were most frequently selected in the total 30 folds were ultimately determined as discriminative radiomic features, including 11 extracted from pre-CRT PET images and 14 from mid-CRT PET images (Table 3), most of which exhibited low correlation with each other (Supplemental materials Table S1). Particularly, higher values of wavelet features extracted from pre-CRT images, such as skewness_HLL, RP_HLL, correlation_HLL, and correlation_LHL, may indicate a higher likelihood of local progression. In addition, patients with larger median SUV, max_LLL, and median_HLL values or a smaller cluster prominence_HLL value extracted from mid-CRT images are more likely to experience local progression (Figs 2 and 3).

**Table 3:** The discriminative radiomic features with the highest AUCs for identifying the local control status of esophageal cancer after CRT.

*Feature*	*AUC* (*median*)	*P-Value* (*median*)
***pre-CRT SUV image:***		
1 correlation	0.73	0.07
2 skewness_LLH	0.75	0.06
3 RP_LLH	0.75	0.51
4 correlation_LHL	0.76	0.02
5 RP_LHL	0.72	0.42
6 kurtosis_HLL	0.74	0.14
7 skewness_HLL	0.77	0.04
8 correlation_HLL	0.76	0.04
9 cluster shade_HLL	0.74	0.52
10 RP_HLL	0.77	0.25
11 LRHGLE_HLL	0.72	0.47
***mid-CRT SUV image:***		
12 mean	0.75	0.07
13 median	0.74	0.09
14 max_LLL	0.77	0.13
15 median_LLL	0.76	0.14
16 max-min_LLL	0.76	0.13
17 autocorrelation_LLL	0.75	0.10
18 sum variance_LLL	0.75	0.11
19 HGLRE_LLL	0.75	0.08
20 SRHGLE_LLL	0.75	0.09
21 LRHGLE_LLL	0.76	0.05
22 correlation_LHL	0.76	0.02
23 median_HLL	0.83	0.03
24 correlation_HLL	0.75	0.04
25 cluster prominence_HLL	0.75	0.04

**Figure 4:**
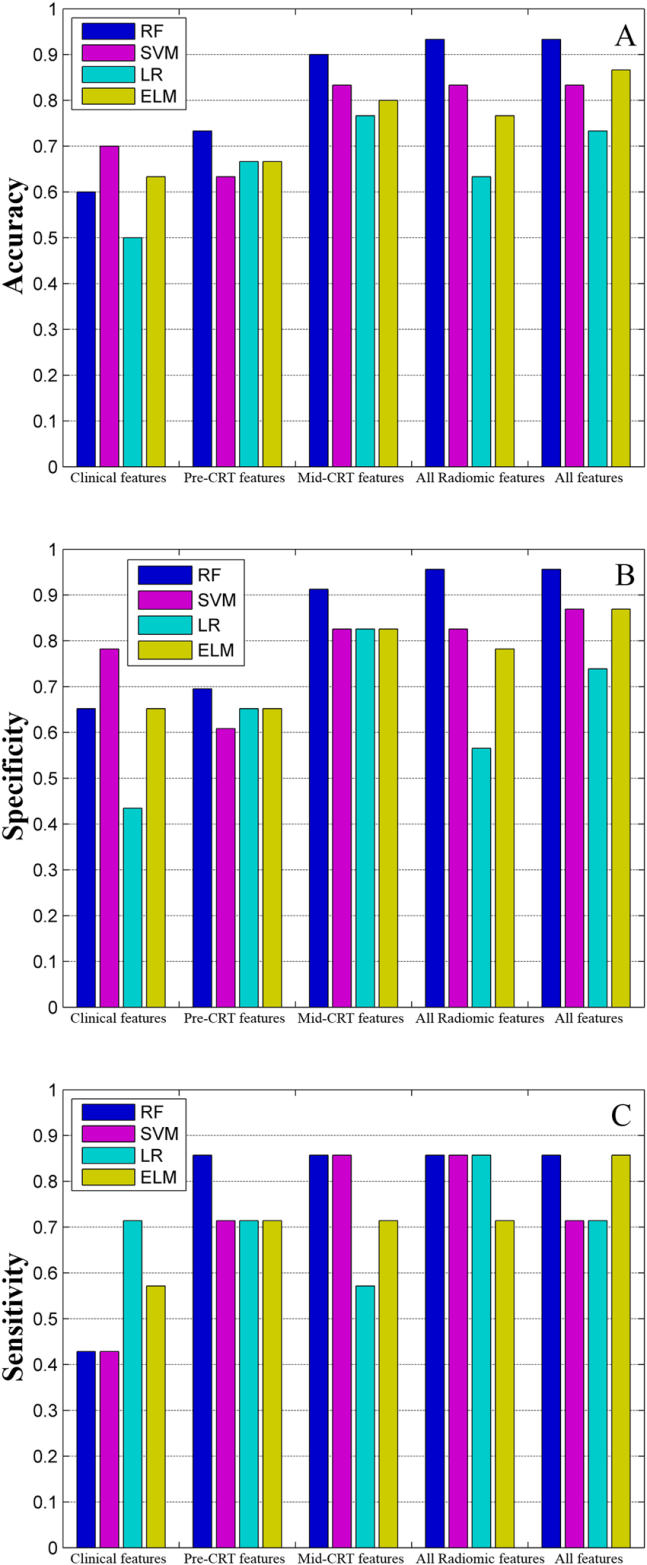
Prediction measures of four models involving specific groups of features. RF, random forest; SVM, support vector machines; LR, logical regression; ELM, extreme learning machine.

Four predicting models (RF, SVM, LR, and ELM) were developed involving specific groups of features, including clinical features, pre-CRT radiomic features, mid-CRT radiomic features, all radiomic features, and a combination of clinical and radiomic features. The prediction performance of the models developed using radiomic features was better than that using clinical features, and better prediction accuracy was achieved by the models developed using mid-CRT radiomic features than that using pre-CRT features, and it was even better when incorporating both pre- and mid-CRT radiomic features. In most cases, the RF model performed better than the SVM, LR, and ELM models when built with the same group of features, except when clinical features alone were involved in the model. The best prediction performance was achieved by the RF model developed with all features, with an accuracy of 93.3%, a specificity of 95.7%, and a sensitivity of 85.7% (Fig. 4). The hyper-parameter values for the RF model involving all features, both the number of samples and candidate features, were set to 10. For the SVM classifier, the penalty factor C and the σ of the RBF kernel were 0.1 and 0.5, respectively. The number of hidden nodes of the ELM with a sigmoidal function was 20.

**Figure 5:**
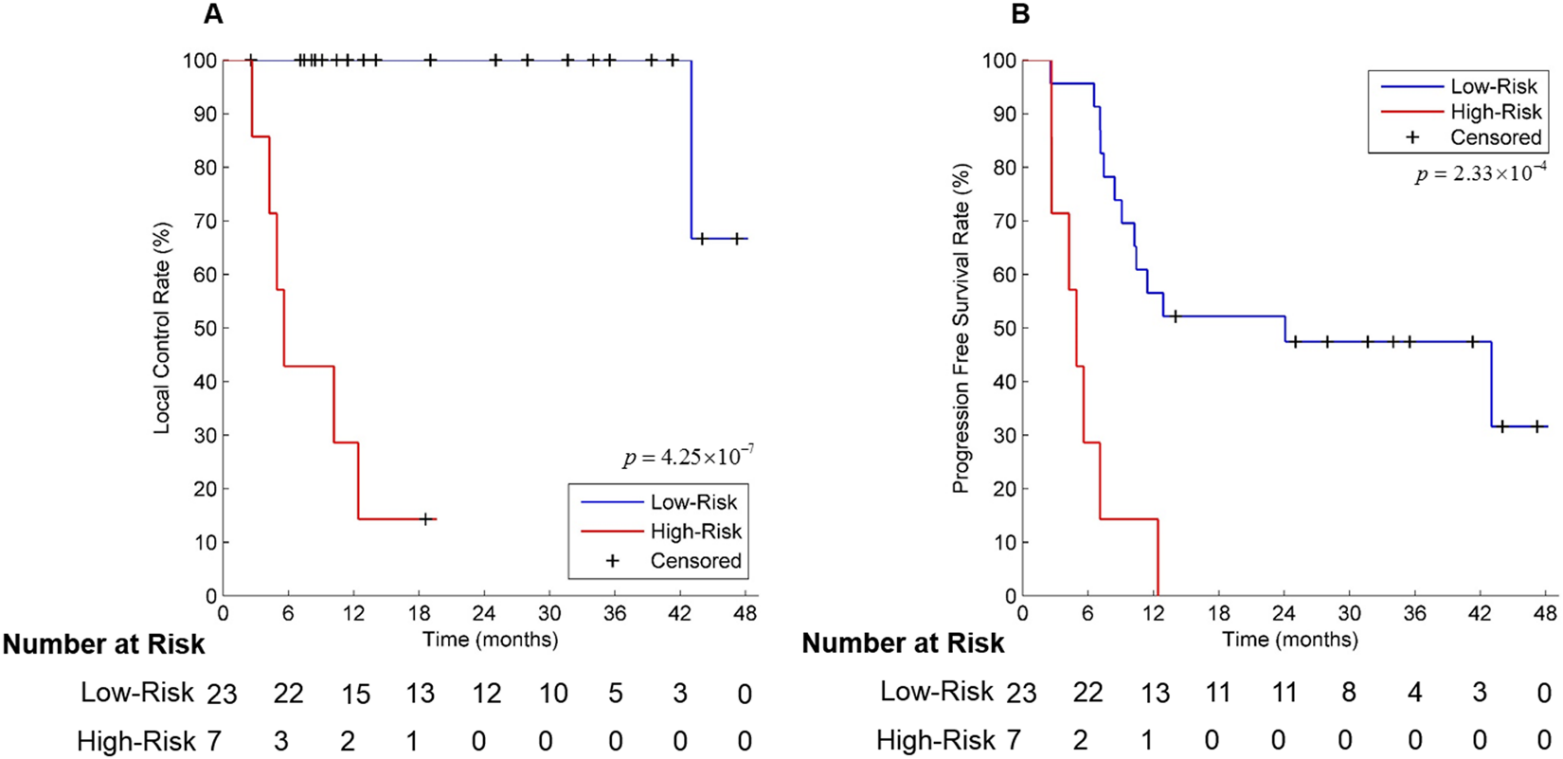
Kaplan-Meier plots of the local control and progression-free survival rates in patients with esophageal cancer treated with CRT based on the risk scores derived from the random forest model incorporating both clinical and radiomic features.

According to the median risk score of local failure estimated by the best predictive model, the patients were classified into two risk groups. The 2-year local control rates were 100.0% (95% CI, 100.0–100.0%) and 14.3% (95% CI, 0.0–40.2%) in the low- and high-risk groups, respectively (p < 0.001, Fig. 5A). The 2-year PFS rates were 52.2% (95% CI, 31.8–72.6%) and 0.0% (95% CI, 0.0–40.2%) in the low- and high-risk groups, respectively (p < 0.001, Fig. 5B). The low-risk group exhibited a significantly prolonged median PFS time compared to that of the high-risk group [18.6 (range, 2.5–47.2) months vs. 5.3 (range, 2.6–12.4) months].

## Discussion

There is an increased number of studies showing the potential of radiomics in identifying tumor phenotypes^19^, enabling tumor diagnosis^12^, predicting the treatment response^6,8^, as well as assessing the tumor prognosis^14^. However, to the best of our knowledge, no effort has been focused on predicting the treatment outcome for unresectable esophageal cancer. Here, we presented an RF model involving both clinical and discriminative radiomic features, which exhibited good performance in predicting the local control status for patients with esophageal cancer treated with CRT. The 25 discriminative radiomic features included 14 mid-CRT features (mean AUC of 0.762) and 11 pre-CRT features (mean AUC of 0.746), indicating that mid-CRT SUV images may be more informative in terms of predicting local control.

Recently, radiomics features extracted from the original images, including first-order statistics, shape- and size-based features^8^ and textural features^12,13^ have been widely investigated. In the present study, we analyzed more radiomics features, especially the wavelet features derived from low- or high-frequency decompositions of the original images. Interestingly, among the selected 25 discriminative features, none were shape- and size-based features, with a maximum AUC value of 0.67 derived from the “surface to volume ratio”. The SUV_median_LLL_ (AUC = 0.76) and SUV_median_HLL_ (AUC = 0.83) derived from wavelet decompositions showed better discriminability than SUV_median_ (AUC = 0.74) derived from first order statistics extracted from the original images. It can be observed that wavelet features predominated among the selected discriminative features (22 out of 25). A possible explanation is that the wavelet transform could overcome and remove most of the noise in low-frequency decompositions and show more details in high-frequency decompositions. Therefore, more robust and abundant features could be acquired from different decompositions than from the original images.

Features in the *correlation* category showed good performance in predicting local control status, including the *correlation* features extracted from both the original images and the wavelet decompositions, as well as those from both the pre-CRT (correlation, correlation_LHL, and correlation_HLL) and the mid-CRT SUV images (correlation_LHL and correlation_HLL). *Correlation* describes the local homogeneity of the input image (the original SUV image or its decompositions). The more similar in intensity a voxel was to its neighbors, the larger the *correlation* would be. The *correlation* would be close to 1 if the intensities of the voxels within a VOI obey Gaussian distribution. It should be noted that the *correlation* feature was computed from the high-frequency decomposition, which represents the details of the original image, such as the peak of a tumor or noise in the image, and has a different physical meaning compared with a *correlation* feature computed from low-frequency decomposition, which represents the rough information of the original image. The good performance of *correlation* suggested the underlying correlation between the homogeneity within the tumor and local tumor control.

The models incorporating radiomic features exhibited better predictive performance than those incorporating conventional clinical features, and no incremental value was observed when clinical features were combined into the model generated with radiomic features, suggesting that radiomic features may provide more information regarding tumor characteristics than clinical features, which have been conventionally used as predictive or prognostic factors in past studies. Furthermore, as shown in Fig. 4, a higher specificity was achieved by the RF model with mid-CRT radiomic features compared to that with pre-CRT radiomic features, which was further improved by combining both pre- and mid-CRT radiomic features; This result indicates that radiomic features extracted from the images during treatment rather than the baseline images may efficiently discriminate local tumor control from other confounding circumstances such as inflammation after treatment or inactivating residual tumor bulk, and such multiple-time point observations would provide more accurate overall judgment for the tumor characteristics associated with treatment.

Compared to the other machine-learning models, SVM, LR, and ELM, the RF model achieved better predictive performance when developed with the same group of features, except for the group of clinical features. The RF is a classification method designed to grow an ensemble of decision trees trained independently on a randomized selection of features. The method performs implicit feature selection when building each independent tree, which can potentially improve the robustness and accuracy of the prediction by combining the merits of inputted features while resisting overfitting. In addition, RF is more robust in handling noise in data and shows better performance in many applications^23,24,29–31^. It is noted that the relative risk score generated from RF, which is different from the one obtained through regression, was just a probabilistic vote assigned to determine whether the patient was going to experience local progression or not. In the present study, the best predictive performance was achieved by the RF model developed with both clinical and radiomic features. Therefore, the risk scores of local failure were generated from this specific model.

Kaplan-Meier analysis exhibited significant separation of both local control curves and PFS curves between the two risk groups. Our results indicated that combining the top 25 radiomic features with RF classification resulted in a promising method to predict the risk of local failure for patients with esophageal cancer who were treated with CRT. This pretreatment and mid-treatment risk estimation and stratification could possibly allow clinicians to deliver more patient-specific treatment tailored to individual risk. It is reasonable that patients with a high risk of local failure could be treated with a dose boost to their GTVs; otherwise, those with a low risk could be prescribed lower doses (60 Gy or less) and be spared of overtreatment. However, whether radiomics-guided patient-specific treatment would ultimately elicit a long-term survival benefit or not requires prospective validation.

In this study, leave-one-out cross validation was used to improve the objectivity of our results. *k*-fold cross validation provides additional information from limited data and prevents the data from falling into the local minimum. A larger *k* corresponds to a more objective result. When *k* equals *n*, the number of samples, *k*-fold cross validation is also called leave-one-out cross validation. Leave-one-out cross validation has two main advantages: first, the model is trained using almost all the samples, with a distribution similar to that of the original set; second, no influence is derived from random factors (e.g., randomly dividing all samples into *k*-fold), which can guarantee the reproducibility of the experiment.

One limitation of our study is that the determination of local control status is much more difficult and may be less accurate for patients who have not undergone surgery. Since the benefit from improved local control may not necessarily translate into improved survival, further investigation is needed to combine radiomics features with other potential factors, such as genomic factors, for better prediction of prognosis. Another limitation is that this was a retrospective analysis of a small sample size. Although leave-one-out cross validation was used to avoid overfitting, independent validation in a larger patient cohort is required to verify the predictive performance of the model developed in the present study.

## Conclusion

The RF model developed with both clinical and 25 PET-based radiomic features resulted in an accurate prediction of local control after CRT in 30 patients with esophageal cancer. Radiomic features acquired from high-frequency decompositions, i.e., wavelet features, and those from multiple pretreatment time points may provide more robust overall information for prediction. However, independent validation in a larger patient cohort is required to confirm the predictive performance of the model. In the future, more prognostic quantitative radiomic features may be explored, and their potential for pretreatment risk stratification may allow clinicians to deliver more patient-specific treatment tailored to individual risk.

## Electronic supplementary material

Supplementary Table S1
