# Supplementary material for: The Role of PET-Based Radiomic Features in Predicting Local Control of Esophageal Cancer Treated with Concurrent Chemoradiotherapy
Source: Sci Rep. 2018 Jul 2;8:9902. doi: 10.1038/s41598-018-28243-x (PMC6028651; doi:10.1038/s41598-018-28243-x)
Supplement: Supplementary file 1 — Supplementary Table S1 [file 41598_2018_28243_MOESM1_ESM.pdf]

The Role of PET-Based Radiomic Features in Predicting Local Control of Esophageal Cancer Treated with Concurrent Chemoradiotherapy

Junfeng Xiong[1,#], Wen Yu[2,#], Jingchen Ma[1], Yacheng Ren[1], Xiaolong Fu[2] and Jun Zhao[1,*]

[1]School of Biomedical Engineering, Shanghai Jiao Tong University, Shanghai, China;

[2]Department of Radiation Oncology, Shanghai Chest Hospital, Shanghai Jiao Tong University, Shanghai, China.

*Corresponding author: Jun Zhao, School of Biomedical Engineering, Shanghai Jiao Tong University, Shanghai, 200240, China (e-mail: junzhao@sjtu.edu.cn).

Junfeng Xiong (e-mail: jfxiong@sjtu.edu.cn)

Wen Yu (e-mail: yuzhiwen0827@163.com)

Jingchen Ma (e-mail: majingchen@sjtu.edu.cn)

Yacheng Ren (e-mail: rycsjtu@sjtu.edu.cn)

Xiaolong Fu (e-mail: xlfu1964@hotmail.com)

# These authors contributed equally in this work.

## Table S1

The Pearson correlation coefficient among the discriminative features.

| **Feature (#)** | **1** | **2** | **3** | **4** | **5** | **6** | **7** | **8** | **9** | **10** | **11** | **12** | **13** | **14** | **15** | **16** | **17** | **18** | **19** | **20** | **21** | **22** | **23** | **24** |
|---|---|---|---|---|---|---|---|---|---|---|---|---|---|---|---|---|---|---|---|---|---|---|---|---|
| **2** | 0.01 | | | | | | | | | | | | | | | | | | | | | | | |
| **3** | 0.44 | 0.12 | | | | | | | | | | | | | | | | | | | | | | |
| **4** | 0.72 | 0.10 | 0.34 | | | | | | | | | | | | | | | | | | | | | |
| **5** | 0.52 | 0.03 | **0.90** | 0.39 | | | | | | | | | | | | | | | | | | | | |
| **6** | 0.11 | 0.24 | 0.12 | 0.13 | 0.09 | | | | | | | | | | | | | | | | | | | |
| **7** | 0.50 | 0.23 | 0.33 | 0.50 | 0.31 | 0.81 | | | | | | | | | | | | | | | | | | |
| **8** | 0.51 | 0.02 | 0.14 | 0.71 | 0.16 | 0.24 | 0.49 | | | | | | | | | | | | | | | | | |
| **9** | 0.07 | 0.09 | 0.29 | 0.06 | 0.25 | 0.55 | 0.37 | 0.02 | | | | | | | | | | | | | | | | |
| **10** | 0.64 | 0.05 | **0.91** | 0.48 | **0.92** | 0.16 | 0.40 | 0.27 | 0.30 | | | | | | | | | | | | | | | |
| **11** | 0.04 | 0.10 | 0.29 | 0.08 | 0.27 | 0.64 | 0.42 | 0.03 | 0.98 | 0.31 | | | | | | | | | | | | | | |
| **12** | 0.02 | 0.28 | 0.08 | 0.06 | 0.07 | 0.15 | 0.11 | 0.07 | 0.22 | 0.03 | 0.24 | | | | | | | | | | | | | |
| **13** | 0.10 | 0.23 | 0.18 | 0.03 | 0.13 | 0.08 | 0.00 | 0.07 | 0.19 | 0.10 | 0.21 | **0.94** | | | | | | | | | | | | |
| **14** | 0.32 | 0.22 | 0.15 | 0.23 | 0.14 | 0.25 | 0.39 | 0.24 | 0.20 | 0.18 | 0.21 | 0.69 | 0.45 | | | | | | | | | | | |
| **15** | 0.05 | 0.18 | 0.14 | 0.09 | 0.08 | 0.02 | 0.00 | 0.15 | 0.10 | 0.05 | 0.11 | **0.90** | **0.95** | 0.44 | | | | | | | | | | |
| **16** | 0.32 | 0.22 | 0.16 | 0.23 | 0.14 | 0.25 | 0.39 | 0.23 | 0.20 | 0.18 | 0.21 | 0.68 | 0.43 | **0.99** | 0.42 | | | | | | | | | |
| **17** | 0.14 | 0.21 | 0.03 | 0.15 | 0.05 | 0.19 | 0.22 | 0.18 | 0.18 | 0.09 | 0.20 | **0.90** | 0.74 | **0.88** | 0.75 | **0.87** | | | | | | | | |
| **18** | 0.13 | 0.21 | 0.03 | 0.15 | 0.05 | 0.19 | 0.22 | 0.18 | 0.18 | 0.09 | 0.20 | **0.89** | 0.72 | **0.88** | 0.73 | **0.88** | **0.99** | | | | | | | |
| **19** | 0.15 | 0.21 | 0.05 | 0.16 | 0.06 | 0.19 | 0.23 | 0.19 | 0.18 | 0.10 | 0.21 | **0.89** | 0.71 | **0.89** | 0.72 | **0.89** | **0.99** | **0.99** | | | | | | |
| **20** | 0.14 | 0.21 | 0.04 | 0.15 | 0.06 | 0.19 | 0.22 | 0.18 | 0.19 | 0.10 | 0.21 | **0.89** | 0.72 | **0.89** | 0.72 | **0.89** | **0.99** | **0.99** | **0.99** | | | | | |
| **21** | 0.17 | 0.17 | 0.08 | 0.18 | 0.06 | 0.20 | 0.25 | 0.24 | 0.17 | 0.12 | 0.19 | **0.82** | 0.63 | **0.90** | 0.65 | **0.89** | **0.97** | **0.97** | **0.98** | **0.98** | | | | |
| **22** | 0.55 | 0.27 | 0.26 | 0.56 | 0.28 | 0.18 | 0.43 | 0.50 | 0.14 | 0.40 | 0.12 | 0.54 | 0.42 | 0.70 | 0.43 | 0.70 | 0.65 | 0.65 | 0.66 | 0.65 | 0.65 | | | |
| **23** | 0.44 | 0.09 | 0.37 | 0.29 | 0.32 | 0.17 | 0.47 | 0.52 | 0.02 | 0.39 | 0.03 | 0.08 | 0.05 | 0.40 | 0.07 | 0.40 | 0.32 | 0.32 | 0.33 | 0.32 | 0.40 | 0.25 | | |
| **24** | 0.46 | 0.14 | 0.23 | 0.39 | 0.22 | 0.22 | 0.39 | 0.40 | 0.27 | 0.33 | 0.26 | 0.77 | 0.67 | 0.77 | 0.68 | 0.76 | **0.82** | **0.81** | **0.82** | **0.82** | **0.81** | 0.76 | 0.41 | |
| **25** | 0.01 | 0.14 | 0.07 | 0.01 | 0.09 | 0.19 | 0.19 | 0.00 | 0.14 | 0.10 | 0.18 | 0.67 | 0.50 | 0.75 | 0.48 | 0.75 | 0.76 | 0.77 | 0.76 | 0.76 | 0.71 | 0.26 | 0.21 | 0.50 |
